# ENU Mutagenesis Identifies Mice with Morbid Obesity and Severe Hyperinsulinemia Caused by a Novel Mutation in Leptin

**DOI:** 10.1371/journal.pone.0015333

**Published:** 2010-12-09

**Authors:** Chen-Jee Hong, Pei-Jane Tsai, Chih-Ya Cheng, Chuan-Kai Chou, Huei-Fen Jheng, You-Chung Chuang, Chia-Ning Yang, Ya-Tzu Lin, Chih-Wei Hsu, Irene H. Cheng, Shiow-Yi Chen, Shih-Jen Tsai, Ying-Jay Liou, Yau-Sheng Tsai

**Affiliations:** 1 Institute of Brain Science, School of Medicine, National Yang-Ming University, Taipei, Taiwan; 2 Division of Psychiatry, School of Medicine, National Yang-Ming University, Taipei, Taiwan; 3 Department of Psychiatry, Taipei Veterans General Hospital, Taipei, Taiwan; 4 Department of Medical Laboratory Science and Biotechnology, College of Medicine, National Cheng Kung University, Tainan, Taiwan; 5 Institute of Clinical Medicine, School of Medicine, National Yang-Ming University, Taipei, Taiwan; 6 National Laboratory Animal Center, National Applied Research Laboratories, Taipei, Taiwan; 7 Institute of Basic Medical Sciences, College of Medicine, National Cheng Kung University, Tainan, Taiwan; 8 Institute of Biotechnology, National University of Kaohsiung, Kaohsiung, Taiwan; 9 Institute of Bioscience and Biotechnology, National Taiwan Ocean University, Keelung, Taiwan; 10 Institute of Clinical Medicine, College of Medicine, National Cheng Kung University, Tainan, Taiwan; University of Padova, Italy

## Abstract

**Background:**

Obesity is a multifactorial disease that arises from complex interactions between genetic predisposition and environmental factors. Leptin is central to the regulation of energy metabolism and control of body weight in mammals.

**Methodology/Principal Findings:**

To better recapitulate the complexity of human obesity syndrome, we applied N-ethyl-N-nitrosourea (ENU) mutagenesis in combination with a set of metabolic assays in screening mice for obesity. Mapping revealed linkage to the chromosome 6 within a region containing mouse *Leptin* gene. Sequencing on the candidate genes identified a novel T-to-A mutation in the third exon of *Leptin* gene, which translates to a V145E amino acid exchange in the leptin propeptide. Homozygous *Leptin^145E/145E^* mutant mice exhibited morbid obesity, accompanied by adipose hypertrophy, energy imbalance, and liver steatosis. This was further associated with severe insulin resistance, hyperinsulinemia, dyslipidemia, and hyperleptinemia, characteristics of human obesity syndrome. Hypothalamic leptin actions in inhibition of orexigenic peptides NPY and AgRP and induction of SOCS1 and SOCS3 were attenuated in *Leptin^145E/145E^* mice. Administration of exogenous wild-type leptin attenuated hyperphagia and body weight increase in *Leptin^145E/145E^* mice. However, mutant V145E leptin coimmunoprecipitated with leptin receptor, suggesting that the V145E mutation does not affect the binding of leptin to its receptor. Molecular modeling predicted that the mutated residue would form hydrogen bond with the adjacent residues, potentially affecting the structure and formation of an active complex with leptin receptor within that region.

**Conclusions/Significance:**

Thus, our evolutionary, structural, and *in vivo* metabolic information suggests the residue 145 as of special function significance. The mouse model harboring leptin V145E mutation will provide new information on the current understanding of leptin biology and novel mouse model for the study of human obesity syndrome.

## Introduction

Leptin, a 16-kDa protein produced mainly in adipose tissue and secreted into the bloodstream, plays an important role in regulating body weight, metabolism and reproductive function [1]. Circulating leptin levels are highly correlated with white adipose tissue mass. The lack of leptin action causes a disruption in energy balance with hyperphagia and decreased energy expenditure, leading to morbid obesity and development of type 2 diabetes. Leptin administration decreases food intake and body weight while preserving metabolic energy utilization. Although obese animals and humans frequently have elevated circulating leptin levels, their leptin fails to mediate weight loss, implicating resistance to the action of leptin in obese states.

Mouse and human leptin cDNA encodes a 167 amino acid residue protein with a 21 amino acid residue signal sequence that is cleaved to yield the 146 amino acid residue mature protein. Mouse leptin shares approximately 96% and 84% sequence identity with the rat and human protein, respectively. Leptin has a four-helix bundle (helices A–D) cytokine structure with an up-up-down-down topology [2], resembling G-CSF and the cytokines of the IL-6 and gp130 family [3].

Hypothalamus is an important site for leptin action, which is mediated by its specific receptor, leptin receptor. Leptin receptor shows highest sequence similarity with the receptors of the IL-6 and gp130 family and the G-CSF receptor [4], [5]. Binding of leptin to leptin receptor in the hypothalamus results in the recruitment and activation of JAK2. Activated JAK2 phosphorylates cytoplasmic domain of leptin receptor, leading to activation and nuclear translocation of STAT3. These in turn modulate transcriptional activity of numerous neuropeptides involved in feeding and energy expenditure, including proopiomelanocortin (POMC), neuropeptide Y (NPY) and agouti-related peptide (AgRP).

Several animal models of monogenic obesity involve mutations in leptin (*Leptin^ob/ob^* mice) or its receptors (*Lepr^db/db^* mice and *fa/fa* rats). The obese phenotype of *Leptin^ob/ob^* mice is caused by mutations in either the coding region (*ob^(1J)^*) [6] or the 5′ non-coding region (*ob^(2J)^*) [7] of mouse *Leptin* gene. Serum leptin level is undetectable in both mutant mice. Mutations in human *LEPTIN* gene have also been reported [8]. Homozygous frameshift (ΔG133) [9] and missense (R105W) [10] mutations of *LEPTIN* gene have been identified in patients with morbid obesity. These mutations result in an inability to produce/secrete the leptin protein, with undetectable levels in the serum of affected individuals. Heterozygosity for leptin mutations is associated with an increase in body weight [11]. In contrast, other leptin missense mutations identified in humans do not appear to influence body weight [12], [13].

To better recapitulate the complexity of human obesity syndrome, we applied a systemic, genome-wide, and phenotype-driven approach in screening of N-ethyl-N-nitrosourea (ENU)-treated mice for obesity. We report here the identification of a novel T-to-A mutation in exon 3 of mouse *Leptin* gene, causing a Val to Glu amino acid exchange in the codon 145 of leptin propeptide. Homozygous mutant mice became obese and hyperphagic at weaning. These obese mice continued to gain weight and develop severe hyperinsulinemia and insulin resistance. In contrast to the absence of immunoreactive leptin protein in the circulation of *Leptin^ob/ob^* mice, homozygous *Leptin^145E/145E^* mice exhibited markedly increased leptin levels in circulation. The mouse model harboring leptin V145E mutation will serve as an excellent model for human obesity which results from leptin dysfunction.

## Results

### ENU-induced obese mice harbor a mutation in leptin

**Figure 1:**
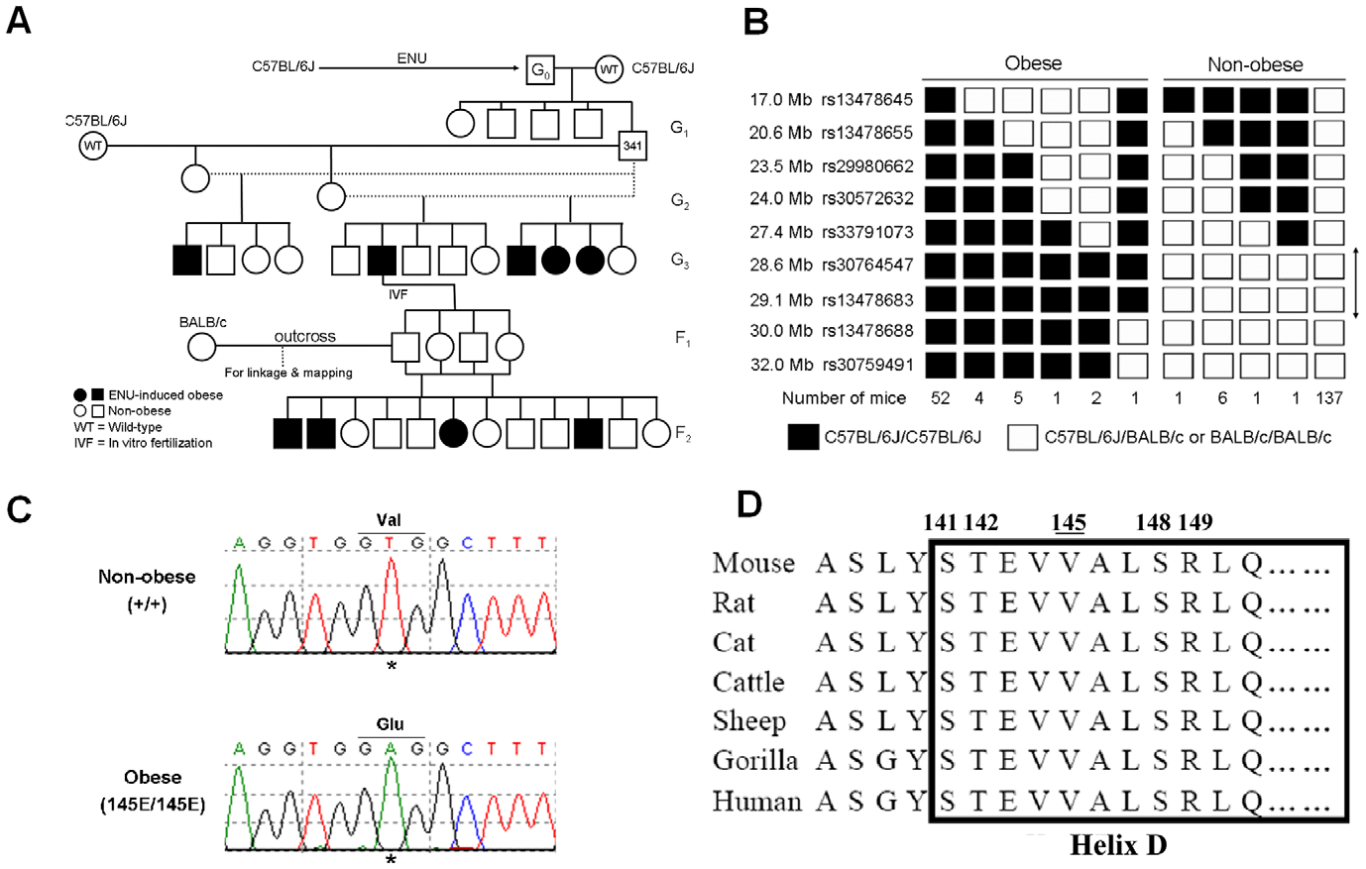
Identification of the leptin mutation in the ENU-induced obese mice. (**A**) Mating and screening strategy for isolation and maintenance of the ENU-induced obese mice. The detail of breeding strategy is summarized in the Materials and Methods. (**B**) Mapping and haplotype analysis for the mutation in the ENU-induced obese mice. Black boxes represent homozygotes for C57BL/6J/C57BL/6J and white boxes represent either C57BL/6J/BALB/c heterozygotes or BALB/c/BALB/c homozygotes. The number of progeny inheriting each haplotype is listed in the bottom. Haplotype analysis indicates that the mutation falls within a 2.6 Mb region (arrow) between rs33791073 and rs13478688 on chromosome 6. (**C**) Sequencing traces a T-to-A transversion (asterisk) in exon 3 of *Leptin* gene, causing a V145E substitution. (**D**) Val-145 and its nearby amino acids, located in the N-terminal of helix D (box) of leptin protein, are conserved throughout species.

During an ENU mutagenesis screen, we found several obese mice from the same G1 founder inherited in a recessive pattern (Figure 1A). Mapping and haplotype analysis established the mutation on chromosome 6 within a 2.6 Mb region between rs33791073 and rs13478688 (Figure 1B). Sequence analysis on the candidate genes within this 2.6 Mb region revealed a T-to-A transversion in exon 3 of *Leptin* gene in homozygous ENU-induced obese mice (Figure 1C). This transversion translates into a Val to Glu amino acid exchange in the codon 145 of propeptide, corresponding to residue 124 of the mature protein. Val-145 and its nearby amino acids, located in the N-terminal domain of helix D of leptin protein, are highly conserved between different species (Figure 1D).

### *Leptin^145E/145E^* mice are obese and hyperphagic

**Figure 2:**
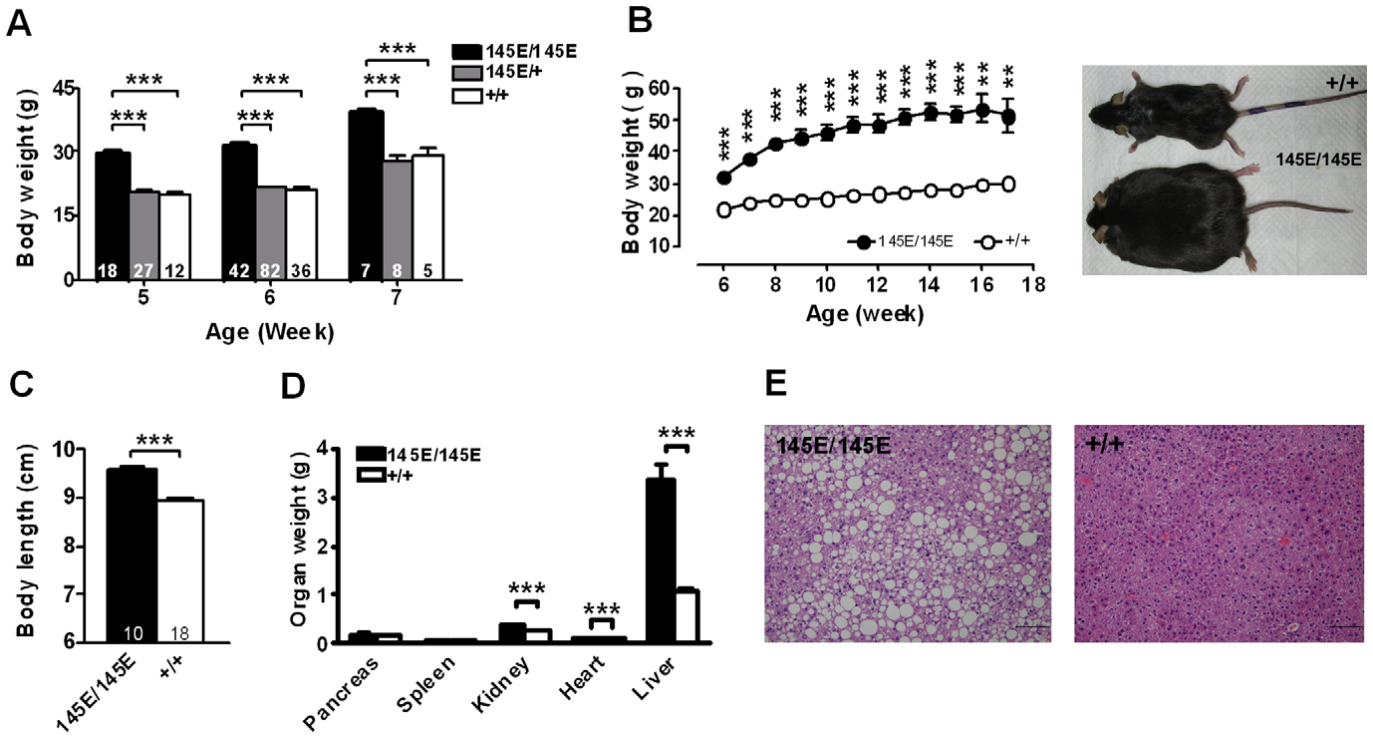
Morbid obesity in *Leptin^145E/145E^* mice. (**A**) Increased body weight in *Leptin^145E/145E^* mice compared with their *Leptin^145E/+^* and *Leptin^+/+^* littermates. (**B**) Growth curves and photograph of male *Leptin^145E/145E^* mice (n = 5) and their *Leptin^+/+^* littermates (n = 9). (**C**) Body lengths of 4-month-old male *Leptin^145E/145E^* and *Leptin^+/+^* mice. (**D**) Organ weights from *Leptin^145E/145E^* (n = 11) and *Leptin^+/+^* mice (n = 19). (**E**) Liver histology of *Leptin^145E/145E^*and *Leptin^+/+^* mice. Numbers of mice are inside bars. **P<0.01 and ***P<0.001 between two genotypes.

Regular chow fed homozygous *Leptin^145E/145E^* mice were extremely obese and infertile. At about 6 weeks of age, body weight of *Leptin^145E/145E^* mice was doubled as those of heterozygous *Leptin^145E/+^* and wild-type *Leptin^+/+^* littermates (Figure 2A). The growth curves of both male and female *Leptin^145E/145E^* mice were steeper than those of *Leptin^+/+^* mice fed regular chow (Figure 2B). Despite the marked increase of body weights in *Leptin^145E/145E^* mice, body lengths of *Leptin^145E/145E^* mice were longer than *Leptin^+/+^* mice by about 7% (Figure 2C). No difference was detectable on pancreas and spleen weights between genotypes (Figure 2D). Kidney and heart weights of *Leptin^145E/145E^* mice were significantly increased, compared with those of *Leptin^+/+^* mice. Liver of *Leptin^145E/145E^* mice were tripled in weight and pale in appearance. Histological analysis identified vacuolization and lipid accumulation in the liver (Figure 2E), suggestive of steatosis in *Leptin^145E/145E^* livers.

**Figure 3:**
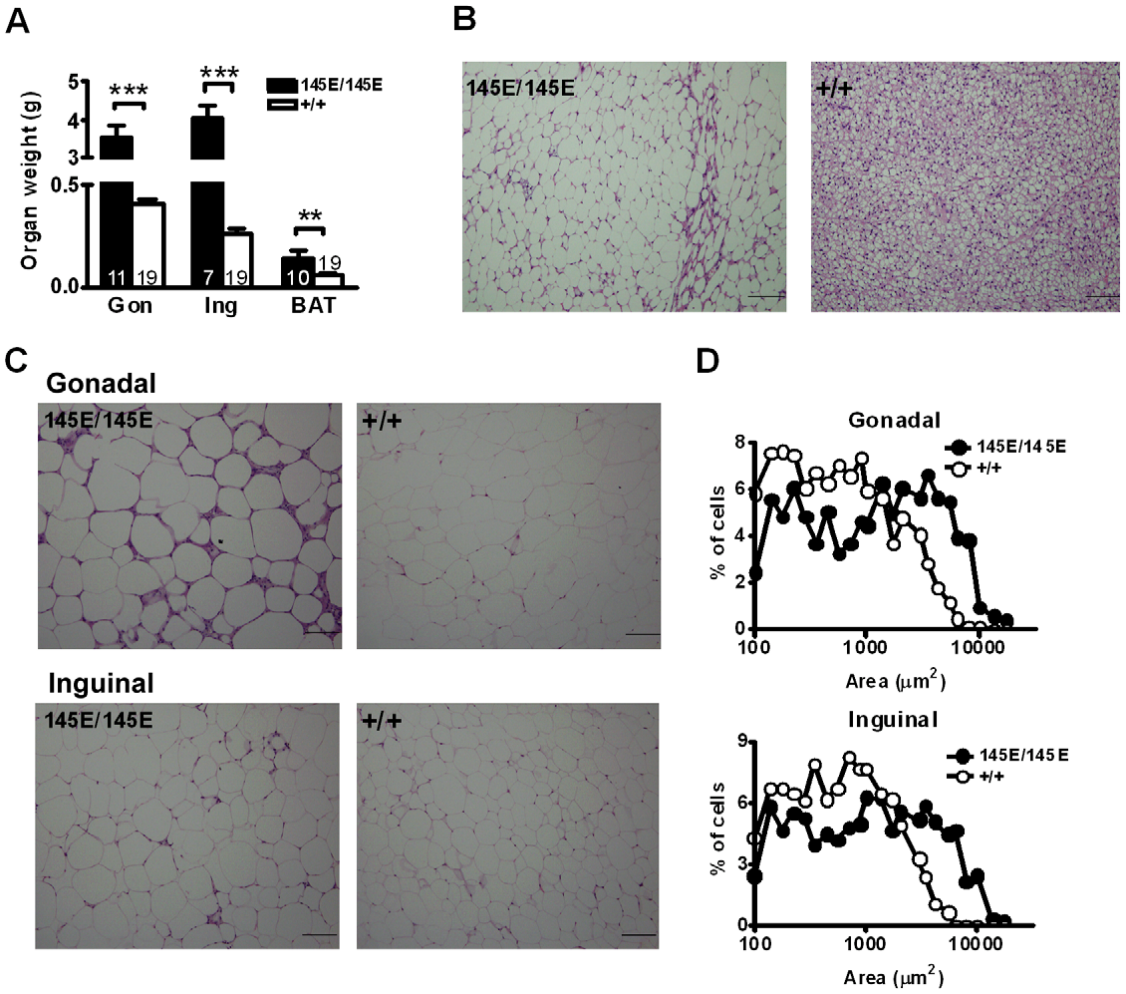
Increased fat deposition in *Leptin^145E/145E^* mice. (**A**) Fat mass of gonadal (Gon) and inguinal (Ing) WAT and interscapular BAT of *Leptin^145E/145E^* (n = 7∼11) and *Leptin^+/+^* mice (n = 19). **P<0.01 and ***P<0.001 against *Leptin^+/+^*. Morphology of (**B**) BAT and (**C**) gonadal and inguinal WAT from 4-month-old male mice. (**D**) Distribution of cell size in gonadal (n = 3 each) and inguinal (n = 3 each) WAT of *Leptin^145E/145E^* and *Leptin^+/+^* mice.

To further analyze the individual fat weight, we dissected gonadal fat to represent intra-abdominal white adipose tissue (WAT), inguinal fat to represent subcutaneous WAT, and interscapular brown adipose tissue (BAT). The gonadal WAT mass in *Leptin^145E/145E^* mice were about 8-fold that of *Leptin^+/+^* mice (Figure 3A). An 18-fold increase in weight was observed in subcutaneous inguinal fat pads of *Leptin^145E/145E^* mice. The interscapular BAT weight of *Leptin^145E/145E^* mice was about 2-fold that of wild-type mice.

Microscopically, adipocytes in BAT exhibited markedly enlarged lipid droplets with unilocular lipid deposition in chow-fed *Leptin^145E/145E^* mice (Figure 3B). Adipocytes in gonadal WAT of *Leptin^145E/145E^* mice were larger than *Leptin^+/+^* cells (mean area 2136 µm^2^ in *Leptin^145E/145E^* versus 897 µm^2^ in *Leptin^+/+^*) (Figure 3C). Similar difference was detected in inguinal WAT of *Leptin^145E/145E^* mice (mean area 2040 µm^2^ in *Leptin^145E/145E^* versus 837 µm^2^ in *Leptin^+/+^*). Thus, the V145E mutation of leptin brought a shift of adipocyte size-distribution toward larger in WAT (Figure 3D). Based on the calculation by DiGirolamo et al. [14], we estimate that the number of adipocytes was doubled in gonadal WAT of *Leptin^145E/145E^* mice (5.1×10^7^ in *Leptin^145E/145E^* versus 2.1×10^7^ in *Leptin^+/+^*). The number of adipocytes in inguinal WAT of *Leptin^145E/145E^* mice (6.2×10^7^) was markedly more than that of *Leptin^+/+^* mice (1.5×10^7^). In addition to adipocyte hypertrophy and hyperplasia, a number of crown-like structures, which reflect the focal convergence of macrophages surrounding necrotic adipocytes, were prevalent in gonadal fat depots of *Leptin^145E/145E^* mice. A marked expansion in cell volume together with the striking increase in cell number thus account for massive increase in fat mass of *Leptin^145E/145E^* mice.

**Figure 4:**
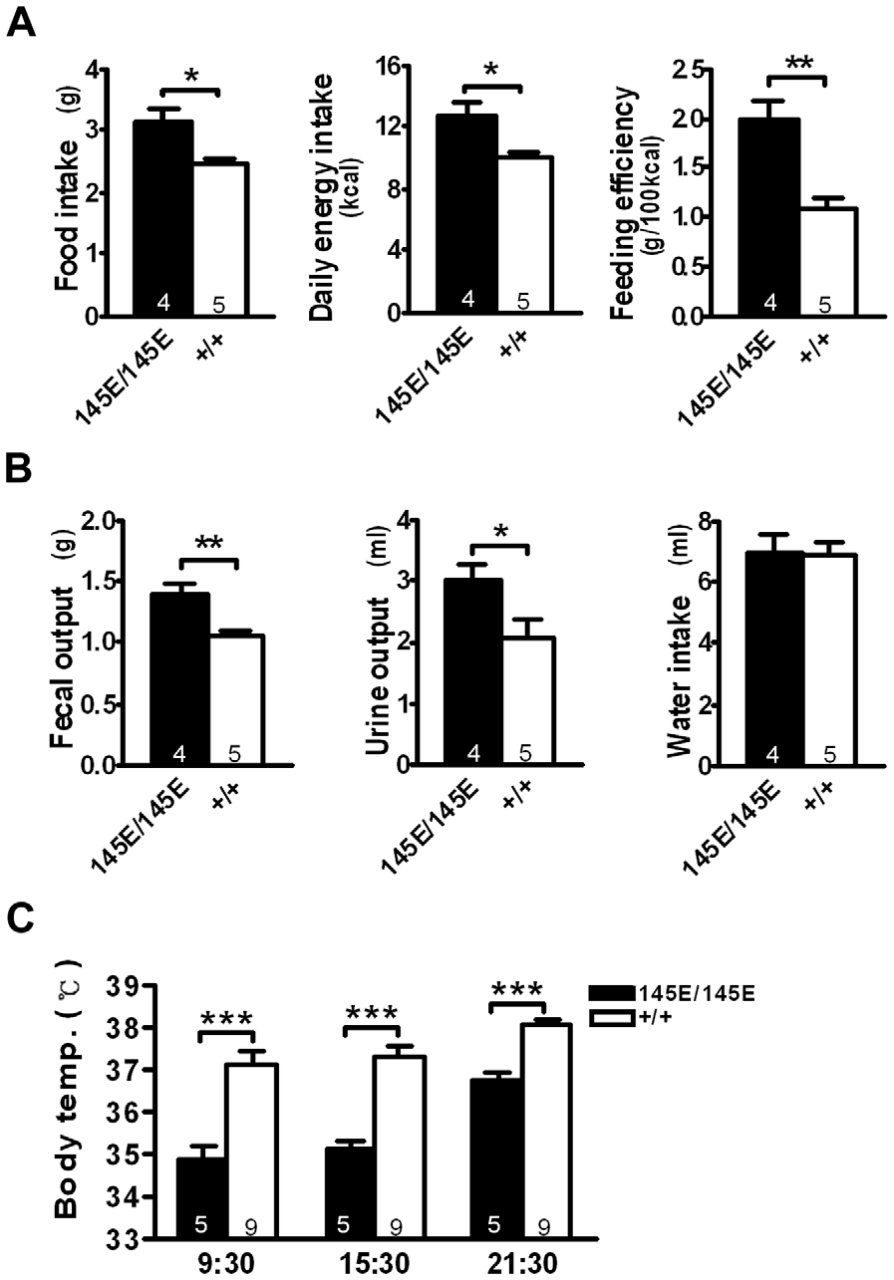
Energy imbalance in *Leptin^145E/145E^* mice. (**A**) Daily food intake, energy intake, and feeding efficiency; (**B**) fecal output, urine output and water intake; (**C**) rectal temperature in *Leptin^145E/145E^* and *Leptin^+/+^* mice. Numbers of mice are inside bars. *P<0.05, **P<0.01, and ***P<0.001 against *Leptin^+/+^*.

*Leptin^145E/145E^* mice had increased daily food intake and caloric absorption compared with those of *Leptin^+/+^* littermates (Figure 4A). Because *Leptin^145E/145E^* mice weighed more than *Leptin^+/+^* mice, the feeding efficiency was significantly increased in *Leptin^145E/145E^* mice. Fecal and urine outputs were slightly increased in *Leptin^145E/145E^* mice (Figure 4B), despite similar water intake between genotypes. In addition, body temperature was significantly decreased in both light and dark cycles (Figure 4C). Thus, *Leptin^145E/145E^* mice exhibited energy imbalance, with increased energy intake and reduced energy dissipation.

### *Leptin^145E/145E^* mice exhibit impaired insulin sensitivity

**Figure 5:**
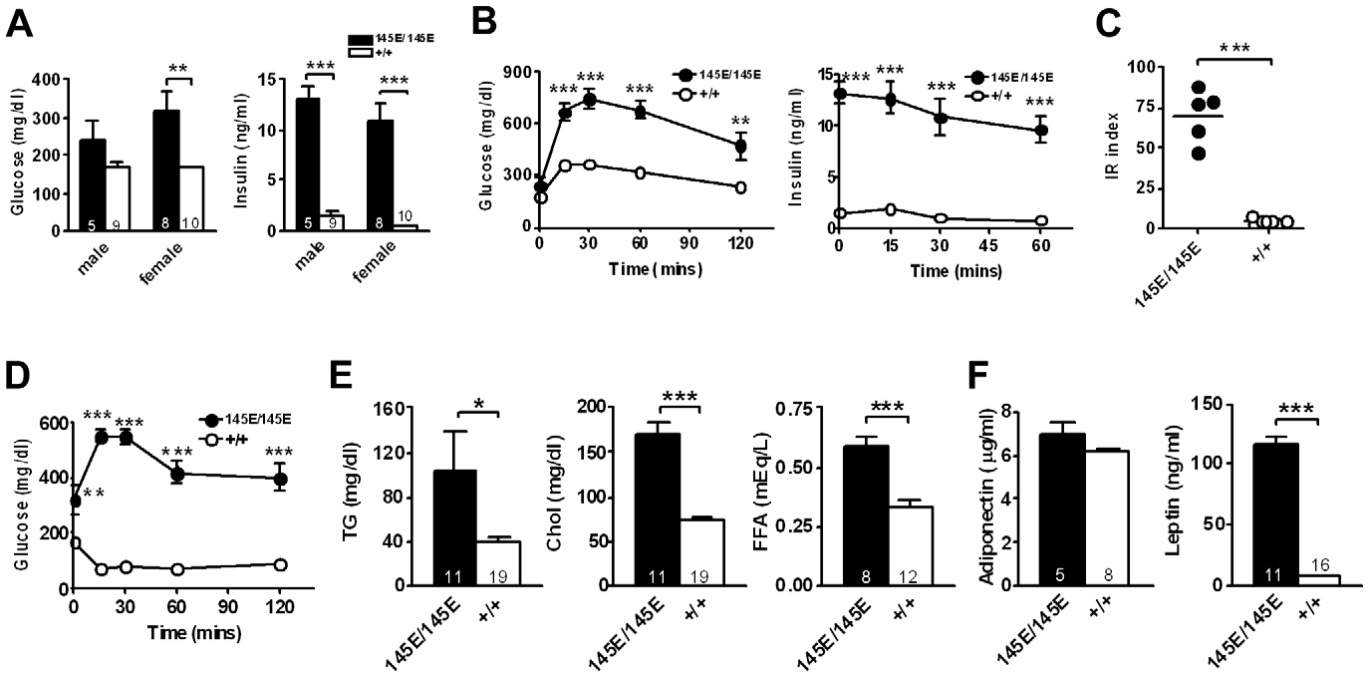
Severe hyperinsulinemia and insulin resistance in *Leptin^145E/145E^* mice. (**A**) Serum glucose and insulin levels during 5-hr fasting of *Leptin^145E/145E^* and *Leptin^+/+^* mice. (**B**) Serum glucose and insulin levels during glucose tolerance tests in 3-month-old male littermates (n = 5∼9). Impaired insulin sensitivity revealed by (**C**) IR index and (**D**) insulin tolerance tests of 3-month-old female *Leptin^145E/145E^* mice (n = 8) compared to *Leptin^+/+^* mice (n = 10). (**E**) Fasting serum triglyceride (TG, left panel), cholesterol (Chol, middle panel), and free fatty acid (FFA, right panel). (**F**) Serum levels of adiponectin (left panel) and leptin (right panel) of *Leptin^145E/145E^* and *Leptin^+/+^* mice. Numbers of mice are inside bars. *P<0.05, **P<0.01, and ***P<0.001 against *Leptin^+/+^*.

To investigate the effect of leptin V145E mutation on glucose homeostasis, we examined the levels of glucose and insulin. The serum glucose and insulin levels after fasting were both significantly higher in *Leptin^145E/145E^* mice than those in *Leptin^+/+^* mice on a regular chow (Figure 5A). To examine the whole-body glucose utilization in *Leptin^145E/145E^* mice, we performed the oral glucose tolerance test (OGTT). *Leptin^145E/145E^* mice cleared glucose less efficiently than *Leptin^+/+^* mice did, indicating glucose intolerance in *Leptin^145E/145E^* mice (Figure 5B). This decreased glucose tolerance was accompanied by significantly increased plasma insulin levels during the OGTT of *Leptin^145E/145E^* mice. Thus, the IR index calculated from the OGTT was markedly increased in *Leptin^145E/145E^* mice (Figure 5C). Consistently, insulin tolerance tests showed that glucose lowering effects of insulin were severely impaired in *Leptin^145E/145E^* mice (Figure 5D). We noticed the initial increase of blood glucose by insulin injection in *Leptin^145E/145E^* mice. The similar observation was demonstrated in other mouse models with severe insulin resistance [15]. Thus, the initial increase of blood glucose by insulin injection in *Leptin^145E/145E^* mice appears to be the consequence of a stress response to handling in the presence of complete insulin unresponsiveness. These results demonstrated that the V145E mutation of leptin impaired glucose tolerance and insulin sensitivity.

Adipose tissues actively secrete signaling molecules, including lipid and adipokines, into the circulation that communicate with other organs to regulate insulin sensitivity [16]. Fasting serum triglyceride and cholesterol levels were significantly higher in *Leptin^145E/145E^* mice than in *Leptin^+/+^* mice (Figure 5E). Although circulating adiponectin levels were not altered, serum leptin levels were dramatically increased in *Leptin^145E/145E^* (Figure 5F). These results imply that the V145E mutation causes the defect in leptin function, leading to a compensatory induction in production of more defective leptin proteins.

### *Leptin^145E/145E^* mice exhibit impaired leptin action in the hypothalamus

**Figure 6:**
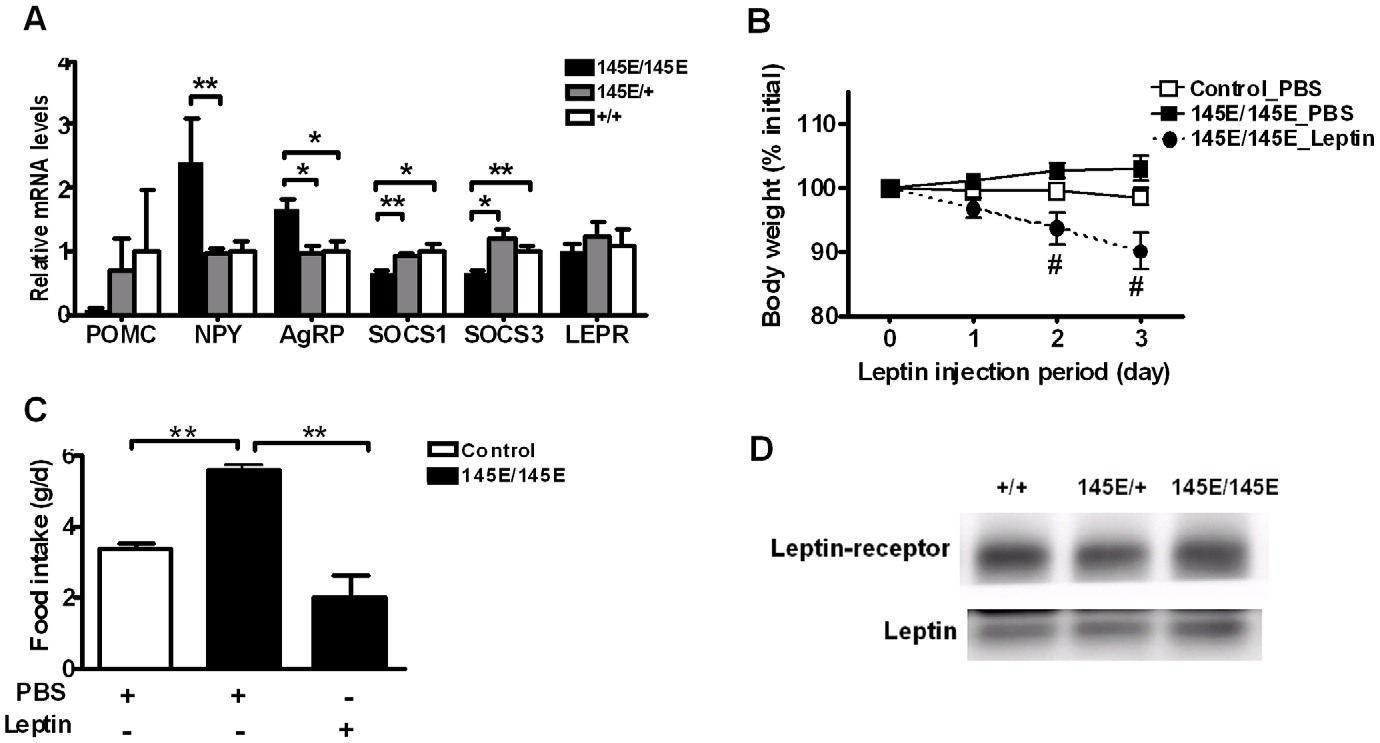
Hypothalamus gene expression and leptin sensitivity in *Leptin^145E/145E^* mice. (**A**) Expression of genes for neuropeptides, including POMC, NPY and AgRP; and SOCS1, SOCS3, and LEPR in the hypothalamus of *Leptin^145E/145E^* mice (n = 5) compared with their *Leptin^145E/+^* (n = 12) and *Leptin^+/+^* (n = 5) littermates. mRNA amount is expressed relative to the average expression in the hypothalamus of *Leptin^+/+^* mice. Leptin sensitivity in *Leptin^145E/145E^* mice revealed by (**B**) body weight and (**C**) daily food intake. *Leptin^145E/145E^* mice (n = 3) and control littermates (including both *Leptin^145E/+^*and *Leptin^+/+^*; n = 3) were injected intraperitoneally twice daily with PBS or leptin (1 mg/kg) for three days. (**B**) Body weight and (**C**) average daily food intake during injection period. Day 0 represents the average weight before leptin injection. *P<0.05 and **P<0.01 between two genotypes/groups. ^#^ *P*<0.05 against PBS-treated *Leptin^145E/145E^* mice. (**D**) Interaction between leptin and leptin receptor in the mid brain was examined by coimmunoprecipitation. Brain lysates from *Leptin^+/+^*, *Leptin^145E/+^* and *Leptin^145E/145E^* mice were immunoprecipitated with anti-leptin receptor antibody, and immunoblotted with anti-leptin or anti-leptin receptor antibodies. Each band represents the tissue extract mixed from three mice.

An important component of leptin action is mediated by the hypothalamus, where leptin promotes expression of anorectic neuropeptide proopiomelanocortin (POMC) and inhibits production of orexigenic neuropeptides neuropeptide Y (NPY) and agouti-related peptide (AgRP) [1]. To determine the effect of V145E mutation on leptin action in the hypothalamus, we examined neuropeptide mRNA expression in hypothalami from mice 8-hour post-feeding, at which circulating leptin levels are raised. While POMC mRNA tended to be lower in *Leptin^145E/145E^* mice than in *Leptin^+/+^* and *Leptin^145E/+^* mice, both NPY and AgRP mRNA levels were significantly increased in *Leptin^145E/145E^* mice (Figure 6A). We also examined mRNA expression for the suppressor of cytokine signaling, SOCS1 and SOCS3, which are known to be induced by leptin [17]. Both SOCS1 and SOCS3 mRNA levels were significantly decreased in *Leptin^145E/145E^* mice (Figure 6A). However, V145E mutation did not affect the expression of leptin receptor (LEPR). Thus, the obese and hyperphagic phenotypes of *Leptin^145E/145E^* mice may result in part from attenuated leptin action in the hypothalamus.

In order to directly access the leptin sensitivity in *Leptin^145E/145E^* mice, we examined the physiological response to low-dose leptin treatment in *Leptin^145E/145E^* mice. Exogenous wild-type leptin administration resulted in a more than 10% decrease in body weight (Figure 6B), and decreased feeding by approximately 60% (Figure 6C). Thus *Leptin^145E/145E^* mice are sensitive to the exogenous administration of wild-type leptin, suggesting the mutant V145E leptin is not functional. To further examine the interaction between mutant V145E leptin and leptin receptor, coimmunoprecipitation analysis was performed from brain using a leptin receptor antibody. Indeed, mutant V145E leptin coimmunoprecipitated with leptin receptor (Figure 6D), suggesting that the V145E mutation does not affect the binding of leptin to its receptor.

### Comparison of metabolic profiles between *Leptin^145E/145E^* and *Leptin^ob/ob^* mice

To compare the metabolic outcome of the V145E mutation in leptin with that of the nonsense mutation at codon 105 of leptin identified in *Leptin^ob/ob^* (*ob^(1J)^*) mice [6], we examined their metabolic parameters in accompany with their respective wild-type littermate controls. We found that *Leptin^145E/145E^* mice have relatively less weight than age-matched *Leptin^ob/ob^* mice, although the weights of their respective wild-type littermate controls were similar (Table 1). Both gonadal and inguinal fat weights relative to their body weights were similar between *Leptin^145E/145E^* and *Leptin^ob/ob^* mice. The ratios of liver to body weights were similar between *Leptin^145E/145E^* and *Leptin^ob/ob^* mice. Although *Leptin^145E/145E^* mice had less body weight, *Leptin^145E/145E^* mice doubled their IR indices compared with age-matched *Leptin^ob/ob^* mice. These results demonstrate that the V145E mutation in leptin results in less adiposity, but more severe insulin resistance, than the truncated premature stop at codon 105 of leptin.

**Table 1:** Comparison of metabolic profiles between *Leptin^145E/145E^* and *Leptin^ob/ob^* mice.

Set of experiment	ENU		ob/ob	
Genotype	*Leptin^145E/145E^*	*Leptin^+/+^*	*Leptin^ob/ob^*	*Leptin^+/+^*
**B.W. (12w)**	48.80±2.79	26.78±0.39	54.19±0.61	25.95±0.51
**B.W. (13w)**	51.00±2.64	27.33±0.45	56.30±0.68	26.18±0.46
**Organ weight (% of B.W.)**				
** Gonadal**	3.57±0.32	0.41±0.03	4.13±0.16	0.38±0.02
** Inguinal**	4.07±0.29	0.27±0.02	3.40±0.10	0.21±0.02
** Liver**	3.36±0.31	1.08±0.04	3.42±0.19	1.33±0.04
**IR index**	70.35±7.24	4.26±0.58	39.45±2.47	5.17±0.35

Organ weights represent % body weights. B.W., body weight.

### Effect of V145E mutation on tertiary structure of leptin

**Figure 7:**
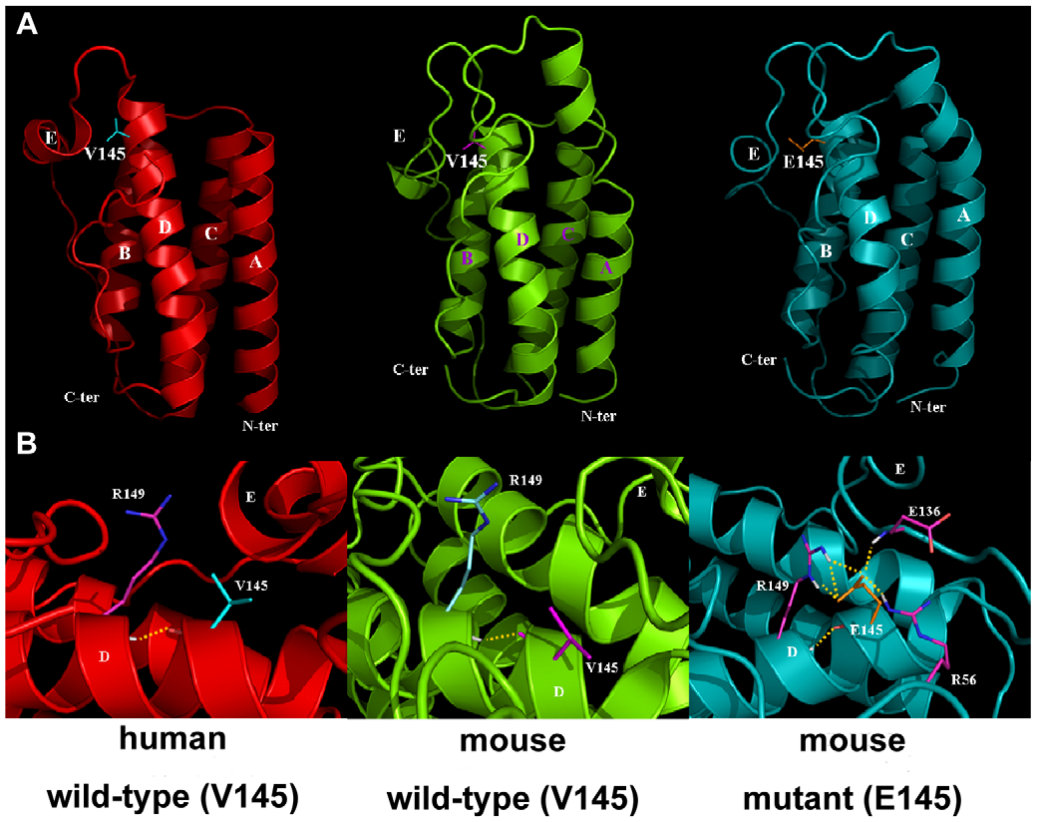
Comparison of tertiary structure of wild-type (V145) and mutant (E145) leptin. (**A**) Tertiary structure of human leptin (left panel) from X-ray crystallography, and wild-type (V145, middle panel) and mutant (E145, right panel) mouse leptins by homology modeling. The four-helical bundle (helices A–D) structure with an additional short helical segment E is shown. The N and C termini are indicated. (**B**) Residue 145 and its adjacent site in wild-type and mutant leptins. The hydrogen bonds between residues are shown as yellow dotted lines. The presented structures were taken from snapshots close to the end of 6 ns simulations.

To examine the effect of V145E mutation on the structure of leptin, we performed the molecular dynamics simulations on wild-type and mutant V145E leptin. Because leptin has high sequence identity among diverse species including human and mouse, the mouse leptin structure was constructed based on the only available X-ray crystal structure of human leptin [2]. Val-145 is located in the N-terminal region of the helix D (Figure 7A). The V145E substitution does not change the overall secondary structure (data not shown) and tertiary structure (Figure 7A). However, a closer inspection on the region of residue 145 indicated that the substitution of valine to glutamic acid with a relatively bulky and positively charged side chain enhances the interstrand interaction through hydrogen bonds formed with the adjacent residues Arg-149, Glu-136 and Arg-56 (Figure 7B). These suggest that the V145E mutation could potentially affect the tertiary structure and formation of an active complex with leptin receptor of this area formed by the residues on helix D, helix E and AB loop.

## Discussion

Using the random ENU-mutagenesis, we created a novel mouse model inherited in a recessive pattern for obesity and insulin resistance due to a missense V145E mutation in the *Leptin* gene. The V145E mutation of leptin leads to a phenotype of extreme obesity, characterized by adipocyte hypertrophy and hyperplasia, positive energy imbalance, and liver steatosis. This was further associated with severe insulin resistance, hyperinsulinemia, dyslipidemia, and hyperleptinemia, characteristics of human obesity syndrome.

In mice, the *Leptin* gene is spontaneously mutated in two independent strains of *Leptin^ob/ob^* mice. In the *ob^(1J)^* allele on the C57BL/6J background, a nonsense mutation at codon 105 (residue 84 of the mature protein) results in the synthesis of a truncated protein that is degraded in the adipocyte [6]. In the *ob^(2J)^* allele on the SM/Ckc-+^DAC^ background, an approximately 5-kb transposon is inserted into the first intron of *Leptin* gene [7]. This mutation results in the synthesis of hybrid RNAs and fails to synthesize mature leptin RNA. To our knowledge, the *Leptin^145E/145E^*mouse model represents the first leptin missense mutation in rodents. The addition of this novel point mutation and its associated phenotype, due to functional alterations in the protein rather than complete lack thereof will prove valuable for understanding the *in vivo* function of leptin. In addition, the failure of elevated leptin levels to mediate weight loss and inhibition of orexigenic neuropeptides suggests the loss of leptin action, and confirms the functional significance of this residue in leptin.

To date, only few *LEPTIN* mutations have been reported in the obese patients. A deletion of a guanidine at codon 133 (ΔG133; corresponding to residue 112 of the mature protein), resulted in the premature stop at position 145, was first reported in two siblings of Pakistani origin [9]. The truncated mutant leptin is misfolded and not secreted. Obese subjects with this mutation express very low or undetectable leptin in the serum. A missense mutation in codon 105 (R105W; residue 84 of the mature protein), that is the same codon mutated in *Leptin^ob/ob^* (*ob^(1J)^*) mice, was uncovered in a Turkish family with morbid obesity [10]. The leptin synthesized in these patients is not secreted in serum. Another missense mutation (N103K) was reported in two Egyptian siblings with severe early onset obesity [18]. However, several missense mutations, including I45V, V110M and E126Q, that do not appear to influence body weight have also been identified [12], [13], [19].

Although three-dimensional structure of leptin was solved [2], the structure of leptin receptor has not yet been determined. The mechanism of leptin-induced leptin receptor activation remains unclear. Our current understanding of molecular interaction of leptin binding to leptin receptor is largely based on homology modeling with other cytokine complex crystal structures [20]. Leptin has been suggested to have three different binding sites I, II, III that interact with leptin receptor [21], [22]. Binding site I, which is thought to be involved in the binding of cytokine receptor homology 1 (CRH1) or CRH2 domain of leptin receptor, appears at the C-terminal region of helix D. Binding site II, which binds to the CRH2 domain of leptin receptor, is composed of residues at the surface of helix A and C. Binding site III, which binds to the immunoglobulin-like domain (IGD) of leptin receptor, is formed by the residues at interface between the N terminus of helix D and the AB loop.

Val-145 resides in the N-terminal region of the helix D (Figure 7A), containing domains important for binding site III of leptin. Val-145 and its surrounding residues (YSTEVVALSRLQ, amino acids 140∼151, black boxed in Figure 1D) are strictly conserved in different species. The substitution of valine to glutamic acid with a sterically larger and positively charged side chain therefore could interfere with relatively hydrophobic environment of the binding site III [23], [24]. This could potentially alter the tertiary structure and formation of an active complex with leptin receptor in this interaction site. Alternatively, the mutated residue Val-145 might be critical for receptor activation by inducing allosteric changes in the receptor upon binding. Finally, we cannot exclude that Val-145 is involved in binding of a yet unidentified domain on leptin receptor.

Although binding site II is thought to be the main high affinity binding site of leptin for leptin receptor [22], [25], [26], binding site III of leptin has been suggested to be responsible for formation of an active multimeric complex and subsequent activation of leptin receptor [5], [27]. Binding site III consists of rather large hydrophobic fragment in the gp130 cytokines [23], [24]. Extensive mutagenesis of mouse and human leptins identified several critical amino acid residues in the N-terminal part of helix D, as main contributors to binding site III. For example, leptin with mutations on Ser-141 and Thr-142 totally lost its ability to activate leptin receptor but showed normal binding to leptin receptor [5]. Moreover, both the mouse and human S141A/T142A leptin mutants exhibit antagonistic activity and block activation of leptin receptor in a dose dependent manner. Another leptin mutation Y140A did not change the binding, but decreased drastically the agonistic activity [27]. Thus mutation located in or close to the binding site III is likely to weaken the interaction with IGD, which would further impair the receptor activation.

Two other leptin mutations, S148D and R149Q, within the N-terminal part of helix D have also been described. Although these two mutations do not affect the binding to the mouse leptin receptor, they cause reduced biological activity [28]. Of particular, R149Q is unable to trigger intracellular signaling and behaves as a competitive inhibitor [29]. Although Arg-149 has not been predicted part of any of the three binding sites, this residue forms hydrogen bonds with the backbone of Pro-64 (in AB loop) and Val-134 (in helix E). Thus, the R149Q mutation has been thought to disturb the proper orientation of the AB loop and helix D, and possibly indirectly affect binding site III [2]. Importantly, the outwardly projecting, positively charged side chain of glutamic acid from mutated reside 145 is predicted to form hydrogen bond with the adjacent residues Arg-149, Glu-136 and Arg-56 (Figure 7B). These suggest that the V145E mutation could potentially affect the role of residue Arg-149 in maintenance of proper environment of binding site III and correct interaction with the receptor. Consistent with the results of leptin mutations on Y140, S141, T142, S148, and R149, the V145E mutation does not appear to affect the binding of leptin to its receptor, despite largely attenuated agonistic activity of leptin.

The adiposity of *Leptin^145E/145E^* mice follows the trend of *Leptin^ob/ob^* mice, although the severity is reduced. The difference was also evident when comparing both genotypes in female mice (data not shown). Despite the difference in adiposity, the percentage of individual fat mass, as well as liver weight, was similar between *Leptin^145E/145E^* and *Leptin^ob/ob^* mice. In contrast, *Leptin^145E/145E^* mice exhibited more impairment in glucose metabolism, reflected in the two-fold increase in IR indices. Because both ENU-induced *Leptin^145E/145E^* and spontaneously-mutated *Leptin^ob/ob^* mice were maintained in C57BL/6, the differences cannot be attributed to their genetic background. Although the phenotypic characterizations of *Leptin^145E/145E^* and *Leptin^ob/ob^* mice were carried out in different sets of experiments, their respective wild-type littermate controls were used in each set of experiment. Based on the similar values between two respective wild-type littermate controls, it is reasonable to compare the magnitude of increases in body fat and insulin resistance. Currently, we cannot exclude the possibility that the obese phenotype in our mutants is accentuated by the concomitant modification of the nearby genes. However, the markedly increased immunoreactive leptin level in circulation suggests a compensatory mechanism to increase the demand for leptin to regulate the energy imbalance. Furthermore, administration of exogenous wild-type leptin attenuated hyperphagia and body weight increase in *Leptin^145E/145E^* mice. Therefore, it is likely that the phenotype is entirely attributable to the nonfunctional V145E leptin protein.

The normal level of adiponectin in *Leptin^145E/145E^* mice is contradicted to the general concept of reduced circulating adiponectin in human obesity syndrome. However, the inconsistency between low circulating adiponectin and obesity/diabetes was also reported in several obese mouse models. For example, a tendency of increase or no difference in plasma adiponectin levels was reported in obese mice with a dominant mutation in *Nmf15* locus [30], *Leptin^ob/ob^* mice [31], and a polygenic obese mouse strain NONcNZO5 [32]. This can partly be explained by the possibility that increased total fat mass in obese mice can temporarily compensate for decreased adiponectin production per unit of fat.

In summary, we successfully used an ENU-based mutagenesis approach in combination with a set of metabolic assays to identify and characterize a novel mouse model with leptin V145E mutation. Two *Leptin^ob/ob^* mouse lines with spontaneously mutated leptin cannot completely model human obesity syndrome because the leptin protein is completely lacking in the serum of those mice. Although residue Val-145 has not been reported to be mutated in humans, our evolutionary, structural, and *in vivo* metabolic information implicates this residue as of special functional significance. Among *LEPTIN* mutations reported thus far in human obesity, ΔG133 and R105W mutations result in an inability to produce/secrete the leptin protein, with undetectable levels in the serum of affected individuals [9], [10]. To our knowledge, the N103K mutation in patients with severe obesity is the only known human mutation that has been demonstrated to disrupt receptor binding [33]. However, our results suggest that the V145E mutation does not affect the binding of leptin to its receptor. Clearly, more studies, such as large-scale genome epidemiology or *in vitro* study using human ES cell systems, are necessary to elaborate correlations between leptin V145E mutation and the severity of obese phenotype in humans. In addition, studies are required to gain further insights on how leptin mutations, not only this V145E mutation, but also other mutations identified in humans, affect the binding to and subsequent activation of leptin receptor. Finally, the V145E substitution in the N-terminus of helix D supplements the known mutations in human and mouse leptin and thereby offers novel mouse model for the study of human obesity syndrome.

## Materials and Methods

### ENU mutagenesis and identification of the mutant

C57BL/6J male mice were treated with ENU (100 mg/kg/week) for 3 weeks [34] at the age of 8 weeks to generate G0 mice. G0 mice were then mated with wild-type female C57BL/6J mice to generate G1 mutagenized founder mice (illustrated in Figure 1A). G1 male mice were mated with wild-type female C57BL/6J mice to generate G2 mice. G2 female mice were backcrossed with G1 male mice to generate G3 mutagenized mice. Several obese mice were found from the same G1 founder (No. 341). One of the obese mice was examined for heredity by a three-generation breeding scheme. Because the obese mice were unable to impregnate the female mice, in vitro fertilization (IVF) was conducted to generate F1 mice which were intercrossed to generate F2 mice. Leptin-deficient (*Leptin^ob/ob^*) mice on a C57BL/6J background were obtained from the Jackson Laboratory. Mice were fed with a regular chow (LabDiet 5P76; PMI Nutrition International, Richmond, IN). Animals were housed in a specific-pathogen-free barrier facility, and handled following procedures approved by the IACUC of Academia Sinica (OMiVGHHC2005108), Taipei Veterans General Hospital (96–160), and National Cheng Kung University (98006).

### Mapping and sequencing for the mutation

Mapping of the mutation was performed using a standard outcross strategy to BALB/c mice in combination with SNP markers specific for the C57BL/6J and BALB/c strains. Seven obese and seven non-obese F2 mice were selected for initial mapping in which 275 SNPs were used. One region located between 17 and 76 Mb of chromosome 6 was found to have complete linkage with obesity. To further narrow the interval, primers and probes flanking the SNPs in the linked region were designed in multiplex format using SpectroDESIGNER software (Sequenom, San Diego, CA). SNP genotyping was performed by high-throughput MALDI-TOF mass spectrometry [35]. Gene mapping analysis was conducted by constructing and comparing figures as shown in Figure 1B. All coding DNA was sequenced using a BigDye dideoxy-terminator system and analyzed on an ABI 3700 sequencer (Applied Biosystems, Foster City, CA). The mutant leptin sequence reported here was deposited in GenBank under accession number HQ166716.

### Metabolic analysis

Daily food intake was examined using metabolic cages (Solo Mouse Metabolic Cage, Tecniplast, Italy). Daily energy intake was calculated from daily food consumed per mouse multiplied by the gross energy for regular chow 4.07 kcal/gm. Feeding efficiency was calculated by dividing the weight increase by their feed consumption over one month of the study [36]. Daily fecal and urine output and water consumption were determined. Rectal temperature was recorded by thermometer (TH-8, Physitemp Instruments, Clifton, NJ).

### Morphological analysis

Paraffin sections (10 µm) of adipose tissues isolated from male mice (n = 3 each) at 16 weeks of age were stained with H&E. Adipocyte size was measured in 500 cells per mouse in several parts of the gonadal and inguinal fat pads using Nikon NIS Elements AR 2.30 software. Mean adipocyte number was determined by dividing the mean fat mass by the mean fat cell size as described previously [14].

### Oral glucose tolerance and intraperitoneal insulin tolerance tests

Mice were fasted for 5 hr and orally gavaged with glucose (2 g/kg body weight) or intraperitoneally injected with human regular insulin (0.35 U/kg body weight, Humulin, Eli Lilly, Indianapolis, IN). Blood samples were collected before and at indicated times after injections. Serum glucose concentration was determined by a glucose colorimetric test (Autokit Glucose, Wako Chemicals USA, Richmond, VA). Insulin was measured using mouse insulin ELISA (Ultrasensitive Mouse Insulin ELISA, Mercodia, Sweden). The IR index was calculated as the product of the areas under glucose and insulin curves in glucose tolerance tests as previously described [37].

### Lipid and adipokine assays

Serum total cholesterol (Cholesterol E, Wako, Osaka, Japan), free fatty acid (NEFA C, Wako, Osaka, Japan) and triglyceride (Stanbio Laboratory, San Antonio, TX) were measured following each protocol. Serum levels of adiponectin and leptin were determined by ELISA (R&D Systems, Minneapolis, MN).

### Tissue collection and RNA analysis

Hypothalami were isolated from 8-hr post fed mice and stored in RNAlater (Ambion, Austin, TX), and RNA was extracted using the TRIzol Reagent (Invitrogen, Carlsbad, CA). mRNA were analyzed with SYBR green-based realtime quantitative RT-PCR (Applied Biosystems, Foster City, CA), with *β-actin* as reference gene in each reaction.

### *In vivo* leptin sensitivity

Mice of 1.5∼3 months of age were intraperitoneally injected twice daily with PBS for initial three days, and then with PBS or murine wild-type leptin (1 mg/kg; PeproTech, Rocky Hill, NJ) for the following consecutive three days. Mice were weighed daily and daily food intake was determined during the injection period.

### Coimmunoprecipitation and protein analysis

Mid brain from mice were isolated and homogenized in RIPA buffer containing protease inhibitors. 500 µg of total protein from each lysate was immunoprecipitated with 10 µg of anti-leptin receptor antibody (ab5593; Abcam, Cambridge, MA) pre-coupled with protein A magnetic beads (LSKM AGA 10; Millipore, Bedford, MA). Immunoprecipitated proteins were separated by 15% SDS-PAGE and transferred to a PVDF membrane. Membranes were incubated with anti-leptin antibody (1∶4000, ab3583; Abcam) or anti-leptin receptor antibody (1∶2000, ab5593; Abcam) and visualized using a chemiluminescence detection system (Millipore).

### Structure modeling

Molecular dynamics simulations on wild-type and mutant mouse leptin was performed based on the X-ray structure of human leptin (PDB entry code: 1ax8) where the AB loop was too flexible to be determined. In our homology modeling to construct the rat leptin, the AB loop was included. The following modeling procedures were used: (1) amino acid residues were added, deleted, or replaced with appropriate residues manually; (2) the structure was energy minimized with Steepest Descent and Conjugate Gradient algorithms. The obtained structure was used for wild-type and for replacing V145 to generate V145E mutant. Both structures were gradually heated to 300 K for 150 ps and additional 6 ns was performed for data collection. The charmM force field was adopted, and time step was set at 2 fs. All the modeling was carried out using the Discovery Studio 2.1 and done with implicit distance-dependent dielectrics solvent model. The SHAKE algorithm was used to constrain all bonds that contain hydrogen atoms; the particle-mesh-Ewald (PME) was introduced for long range electrostatic interactions.

### Data analysis

Values are reported as mean ± s.e.m.. Statistical analysis was conducted by multifactorial ANOVA with genotype, gender, and age as factors. Student's *t*-test was used for comparisons between mutant and wild-type mice within each group, and differences were considered to be statistically significant at *P*<0.05.
